# Specifications of ZnO growth for heterostructure solar cell and PC1D based simulations

**DOI:** 10.1016/j.dib.2015.09.050

**Published:** 2015-10-14

**Authors:** Babar Hussain, Abasifreke Ebong

**Affiliations:** Department of Electrical and Computer Engineering, University of North Carolina at Charlotte, USA

**Keywords:** Zinc oxide, Silicon, Solar cell, Antireflection coating, Heterojunction, PC1D, MOCVD

## Abstract

This data article is related to our recently published article (Hussain et al., in press [1]) where we have proposed a new solar cell model based on n-ZnO as front layer and p-Si as rear region. The ZnO layer will act as an active n-layer as well as antireflection (AR) coating saving considerable processing cost. There are several reports presenting use of ZnO as window/antireflection coating in solar cells (Mansoor et al., 2015; Haq et al., 2014; Hussain et al., 2014; Matsui et al., 2014; Ding et al., 2014 [2], [3], [4], [5], [6]) but, here, we provide data specifically related to simultaneous use of ZnO as n-layer and AR coating. Apart from the information we already published, we provide additional data related to growth of ZnO (with and without Ga incorporation) layers using MOCVD. The data related to PC1D based simulation of internal and external quantum efficiencies with and without antireflection effects of ZnO as well as the effects of doping level in p-Si on current–voltage characteristics have been provided.

**Specifications table**

**Table:** 

Subject area	Physics
More specific subject area	Solar cells
Type of data	Figures, and optical images
How data was acquired	1. PC1D simulations 2. Photoluminescence measurements by MiniPL-5.0 system by Photon Systems Inc., USA 3. Ex-situ thickness and transmission measurements by Filmetrics-205-0509, USA 4. In-situ reflectance measurements by Filmetrics-205-0034, USA
Data format	Analyzed
Experimental factors	Before ZnO growth by MOCVD, substrates were cleaned with acetone, methanol, and isopropanol subsequently and dried up with nitrogen in clean room
Experimental features	The precursors used for ZnO growth are diethylzinc (DEZn) and pure oxygen (O_2_) where nitrogen (N_2_) was used as a carrier and dilution gas. The reactor pressure and susceptor rotation speed were kept constant at 4 Torr and 800 rpm respectively. The flow rates of oxygen and carrier gas were 1000 and 100 sccm respectively. The bubbler pressure during growth stayed constant around 180 Torr. The bubbler temperature was kept at 5 °C resulting in vapor pressure of DEZn about 5 Torr which resulted in VI/II ratio around 330 [1].
Data source location	Charlotte, USA Latitude: 35.305373, Longitude: −80.730964
Data accessibility	Data is with this article and in Ref. [1]

## 1 Value of the data

• The specifications for ZnO growth using MOCVD help preparing ZnO films as front n-layer of the solar cell with improved transparency.
• The PC1D simulations give a good explanation of optimization of parameters. The researchers interested in fabrication of the proposed solar cell do not need to do iterative experiments to optimize doping level in absorber (p-Si).
• For the researchers working in ZnO growth using MOCVD (for example [2], [3], [4], [5], [6]), the optical pictures of reactor from inside provided in this article give an idea of dynamics of the MOCVD reactor we used. It will help them to compare differences in material quality of the device.

## 2 Data, experimental design, materials and methods

**Fig. 1:**
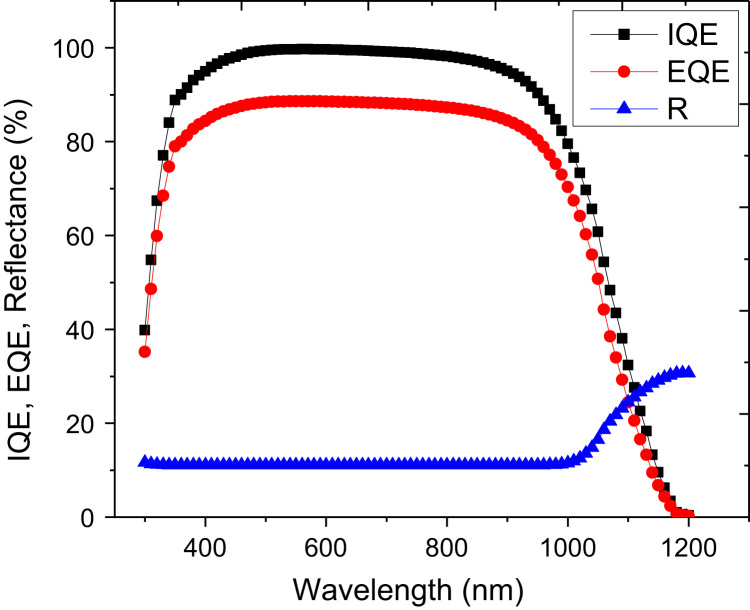
Internal quantum efficiency (IQE), external quantum efficiency (EQE), and front surface reflection (R) of the solar cell device. The effects are shown without taking into account antireflection effects of the ZnO layer.

**Fig. 2:**
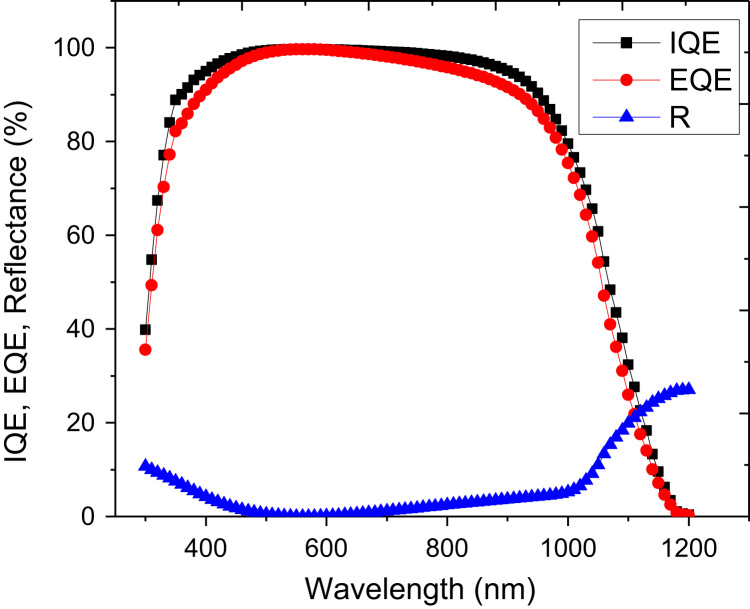
Internal quantum efficiency (IQE), external quantum efficiency (EQE), and front surface reflection (R) of the solar cell device taking into account antireflection effects of the ZnO layer.

There are several adjustable parameters in PC1D which can be iterated to find an optimized window for solar cell fabrication. Since we are using ZnO only for the front region, the parameters associated with the rear region are almost same as already optimized for Si by the solar cells community. We have used absorption spectrum for ZnO which was measured in our lab for film thickness of ~500 nm. Fig. 1 illustrates internal quantum efficiency (IQE), external quantum efficiency (EQE), and front surface reflection of the solar cell device. The antireflection effects of the ZnO layer were not considered for this simulation. The reflectance and quantum efficiency with incorporation of antireflection in device parameters are depicted in Fig. 2. It is obvious that absorption as well as EQE is significantly improved specially around wavelength of 600 nm (peak of solar spectrum).

**Fig. 3:**
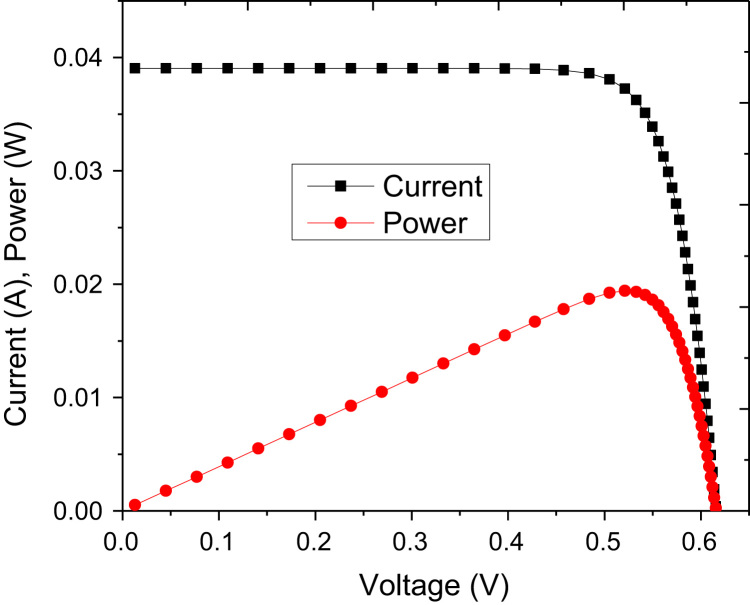
Current, voltage, and power characteristics of the Si–ZnO single heterojunction solar cell with optimized parameters by simulations.

**Fig. 4:**
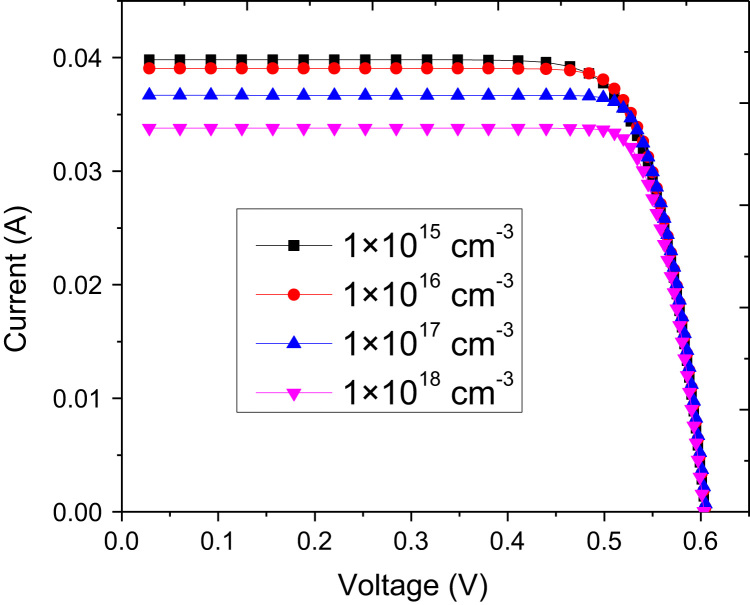
Current voltage characteristics of the solar cell with different doping concentrations in p-Si, keeping n-ZnO doping concentration constant at 2.2×10^19^ cm^−3^.

The current–voltage (*I*–*V*) and power characteristics of the device are shown in Fig. 3 for optimized parameters. The best conversion efficiency achieved was 17.6% with fill factor of 0.808. These values are computed without antireflection incorporation. Integrating antireflection in the simulation increased the conversion efficiency to 19% with almost same value of fill factor. The doping concentration in ZnO has significant influence on the fill factor. The fill factor reduced quickly for concentrations lower than that of order 10^17^ cm^−3^ as reported elsewhere [7]. Change in ZnO doping concentration does not change short circuit current (*I*_SC_) and open circuit voltage (*V*_OC_) significantly. The doping concentration in Si does not alter fill factor and *V*_OC_ prominently but it changes *I*_SC_ significantly as illustrated in Fig. 4. It can be noted that *I*_SC_ reduces with increasing p-doping concentration in Si.

**Fig. 5:**
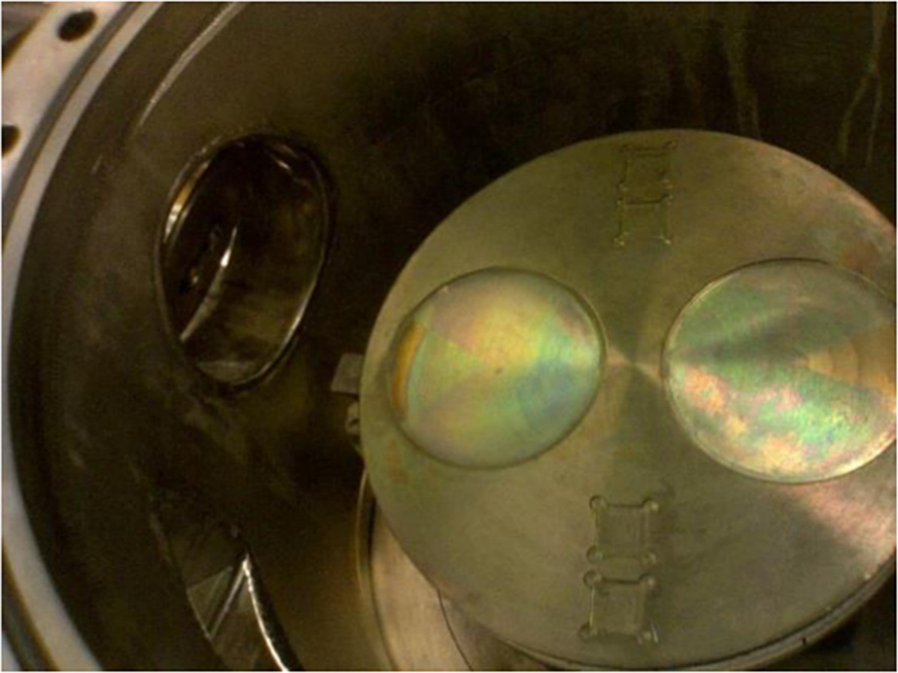
Optical image of inside of the MOCVD reactor.

**Fig. 6:**
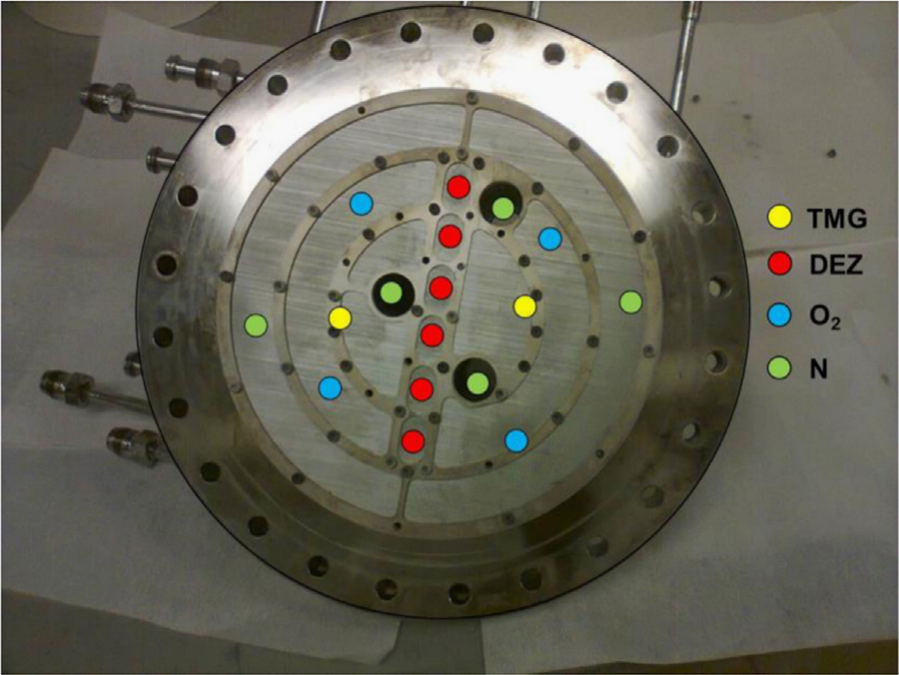
Distribution of gas flows through shower head of the MOCVD reactor.

A homemade MOCVD system was used to grow ZnO films on Saphhire substrates using previously optimized parameters [8], [9] at a range of growth temperature. The cleaned substrates were placed in one of the grooves of the susceptor as shown in Fig. 5. The distribution of gas flows through shower head of the reactor is depicted in Fig. 6. Details about the film growth and characterization across the film surface is reported somewhere else [9], [10]. Detailed optical characterization based on Raman spectroscopy can be found in other articles [11], [12], [13], [14], [15]. Further work is in progress in our labs to improve uniformity in film quality across the surface and growth/characterization of ZnO films on p-silicon to fabricate the proposed solar cell device [1].
