# Corrigendum to “Specifications of ZnO growth for heterostructure solar cell and PC1D based simulations” [Data Brief 5 (2015) 516–521]

**DOI:** 10.1016/j.dib.2016.03.034

**Published:** 2016-03-14

**Authors:** Babar Hussain, Abasifreke Ebong

**Affiliations:** Department of Electrical and Computer Engineering, University of North Carolina at Charlotte, USA

The authors regret “My current advisor and second author of the paper Dr. Abasifreke Ebong thinks that we should add the name of my ex-advisor Dr. Ian Ferguson as third author and I agreed with Dr. Ebong”.

The authors would like to apologise for any inconvenience caused.

